# Effects of lid debris debridement combined with meibomian gland expression on the ocular surface MMP-9 levels and clinical outcomes in moderate and severe meibomian gland dysfunction

**DOI:** 10.1186/s12886-021-01926-2

**Published:** 2021-04-12

**Authors:** Su Young Moon, Sol Ah Han, Hye Ji Kwon, So Young Park, Jae Hyuck Lee, Ho Seok Chung, Jae Yong Kim, Hungwon Tchah, Hun Lee

**Affiliations:** Department of Ophthalmology, Asan Medical Center, University of Ulsan College of Medicine, 88 Olympic-Ro 43-Gil, Songpa-Gu, Seoul, 05505 South Korea

**Keywords:** Dry eye, Lid debris debridement, Matrix metalloproteinase-9, Matrix metalloproteinase-9 immunoassay device, Meibomian gland dysfunction, Meibomian gland expression

## Abstract

**Background:**

To evaluate the effects of lid debris debridement and meibomian gland expression (MGX) on extracellular matrix metalloproteinase-9 (MMP-9) levels and clinical outcomes of moderate and severe MGD.

**Methods:**

In this retrospective case series study, a total 48 eyes of 24 patients with moderate and severe MGD underwent one session of lid debris debridement using the BlephEx combined with MGX. We evaluated the tear film break-up time (TBUT), corneal and conjunctival fluorescein staining scores, Schirmer 1 test, biomicroscopic examination of lid margins and meibomian gland (MG), ocular surface disease index (OSDI) questionnaire score, and extracellular MMP-9 levels using a point-of-care MMP-9 immunoassay device before and 4 weeks after lid debris debridement and MGX. Linear mixed model and generalized estimating equations model were used to evaluate possible differences.

**Results:**

There were significant improvements in the TBUT (*P* = 0.002), SICCA and Oxford staining scores (all *P* < 0.001), lid margin telangiectasia (*P* < 0.001 for upper and lower eyelids), lid thickness (*P* < 0.001 for upper and lower eyelids), MG orifice plugging (*P* < 0.001 for upper and lower eyelids), meibum color (*P* = 0.026 for upper eyelid, *P* < 0.001 for lower eyelid), meibum consistency (*P* < 0.001 for upper and lower eyelids), meibum grade (*P* < 0.001), MGD stage (*P* < 0.001), and OSDI score (*P* = 0.002). MMP-9 immunoassay positivity rate significantly decreased from 83.3 to 50.0% 4 weeks after treatment (*P* = 0.014).

**Conclusions:**

In patients with moderate to severe MGD, lid debris debridement using the BlephEx combined with MGX improved clinical findings, subjective symptoms, meibomian gland function, along with ocular surface MMP-9 level. We hereby suggest lid debris debridement using BlephEx combined with MGX as an effective clinical strategy for treatment of moderate to severe MGD.

## Background

Dry eye disease, despite its frequent diagnosis in ophthalmology, is often intractable despite diverse treatment efforts of ophthalmologists. Patients report various ocular symptoms caused by tear film instability, hyperosmolarity, ocular surface inflammation, and neurosensory abnormalities [1]. Meibomian gland dysfunction (MGD) is a prevalent condition and a major cause of dry eye [2]. MGD is commonly characterized by terminal duct obstruction and changes in lipid-based glandular secretion, resulting in a deficient outer protective layer of the tear film, and is generally associated with a chronic course with intermittent flares [3–5].

The goal of MGD treatment is to provide long-term alleviation of the symptoms for patients by improving the quality of the meibum and tear film stability, and by decreasing ocular surface inflammation. Conventional eyelid management is not sufficient to resolve inspissated meibum [6]. Thus, additional measures are needed to relieve obstructed meibomian gland efficiently and to modulate subsequent inflammatory processes in moderate and severe MGD [7, 8]. Systemic tetracycline, doxycycline, minocycline, and topical ophthalmic solutions, such as azithromycin, loteprednol etabonate, cyclosporine, and diquafosol, can be effective when prescribed concurrently with conventional eyelid management [7–11]. Recently, several studies have reported the efficacy of intensive pulsed light (IPL) treatment for MGD, even for the refractory type [11–17].

Lid debris debridement using the BlephEx system (RySurg, Fort Worth, FL, USA) is a novel form of eyelid management that removes the accumulated bacterial biofilm from the lid margin and scurf from the eyelashes [18]. However, to the best of our knowledge, previous studies have not evaluated the effect of mechanical lid debridement on ocular surface inflammation using the matrix metalloproteinase-9 (MMP-9) immunoassay device (RPS InflammaDry; Rapid Pathogen Screening Inc., Sarasota, FL, USA) to the assess extracellular MMP-9. Therefore, this study aims to investigate the effect of lid debris debridement by measuring the extracellular MMP-9 levels using an MMP-9 immunoassay device and by evaluating the change in clinical findings, subjective symptoms, and meibomian gland function in patients with moderate to severe MGD.

## Methods

This study was approved by the Institutional Review Board of the Asan Medical Center and the University of Ulsan College of Medicine, Seoul, South Korea (2020-0096) and performed in accordance with the Declaration of Helsinki and relevant guidelines.

Patients diagnosed with moderate and severe MGD at Asan Medical Center IPL Dry Eye Clinic who met the inclusion criteria were enrolled in the study. Patients had to be at least 20 years old. Moderate and severe MGD was diagnosed based on tarsal conjunctival erythema, bulbar conjunctival hyperemia, telangiectasia, thickening and irregularity of the eyelid margin, and meibomian gland orifice inclusions. MGD staging was determined using the criteria stated in the International Workshop on Meibomian Gland Dysfunction described by the Tear Film and Ocular Surface Society. Patients with moderate degree of symptoms including ocular discomfort, itching or photophobia, moderate degree of clinical signs (plugging, telangiectasia, moderately altered secretions from grade 8 to less than 13, expressibility 2), and mild to moderate conjunctival and peripheral corneal staining were diagnosed with moderate MGD. Diagnosis of severe MGD was made according to following criteria: patient report of considerable ocular discomfort, itching or photophobia, office-based clinical assessment (dropout, displacement, severely altered secretions of grade 13 or greater, expressibility 3), notable degree of conjunctival and corneal staining or clinical indications of elevated inflammation, including but not limited to moderate conjunctival hyperemia, phlyctenules [19, 20]. The exclusion criteria are as follows: history of previous ocular surgery, diagnosis of glaucoma or ocular hypertension, clinical sign of active ocular infection or non-dry eye ocular inflammation, ocular allergy, diagnosis of autoimmune disease, use of contact lenses during the study period, presence of punctal occlusion, use of anti-inflammatory eye-drops and oral antibiotics within a month before enrollment, active skin lesions or previously diagnosed skin pathology, ocular rosacea, pregnancy or lactation.

### Measurements

Clinical evaluation was performed before initiating the treatment and 4 weeks after the treatment. The parameters used for clinical examination include extracellular MMP-9 levels using the MMP-9 immunoassay device, tear film break-up time (TBUT), corneal and conjunctival staining score (Sjögren’s International Collaborative Clinical Alliance [SICCA] ocular staining and Oxford staining score), Schirmer 1 test performed without topical anesthetics, lid margin abnormalities and meibum grade [21–24].

The MMP-9 level of the ocular surface was measured using the MMP-9 immunoassay device, which is a point-of-care test that analyzes the tear samples collected in the office [25]. Existence of one blue line and one red line in the MMP-9 test result window means a positive test result (MMP-9 ≥ 40 ng/mL: strong positive, positive, and weak positive), while single blue line means a negative test result (MMP-9 < 40 ng/mL: trace and negative) [24]. We also measured the MMP-9 level based on the change in the intensity of color at 10 min using standardized photographs as a reference on a scale of 0 to 4: 0, none; 1, trace; 2, weak positive; 3, positive; and 4, strong positive [24].

A single sterile sodium fluorescein strip was wet with non-preserved saline solution then touched to the bulbar conjunctiva to measure TBUT. TBUT was defined as the time lapse between a blink and appearance of the first spot on the corneal surface and the average of 3 measurements was taken. Corneal and conjunctival fluorescein staining was examined, which was followed by Schirmer 1 test (without topical anesthetic). Fluorescein staining was graded according to the SICCA and Oxford scale.

The lid margin was examined under slit-lamp microscope and recorded separately for upper and lower lids. We assessed telangiectasia (on a scale of 0–3, with 0 = no findings; 1 = mild telangiectasia; 2 = moderate telangiectasia or redness; and 3 = severe telangiectasia or redness), anterior or posterior replacement of the mucocutaneous junction, and lid abnormalities, including irregularity, thickness, and plugging (on a scale of 0–2, with 0 = no finding; 1 = mild; and 2 = severe) [21–23]. To assess the meibum grade, the degree of digital pressure applied on the upper tarsus during MG expression was recorded by the physician, the grading was as follows: grade 0 = clear meibum easily expressed; grade 1 = cloudy meibum expressed with mild pressure; grade 2 = cloudy meibum expressed with more than moderate pressure; and grade 3 = no meibum expression, even with firm pressure [26]. The color (on a scale of 1–3, 1 = clear; 2 = cloudy; and 3 = yellow) and consistency of expressed meibum (on a scale of 1–3, 1 = oily; 2 = creamy; and 3 = toothpaste like) were assessed for both upper and lower eyelids using the meibomian gland expressor forceps [21].

Patients self-reported their symptoms using the ocular surface disease index (OSDI) questionnaire and ocular irritation symptom (ocular discomfort, itching, and photophobia with limitations of activities) and the severity was graded on a scale of 0 (no symptom) to 3 (severe symptom). In order to minimize its effect on each other, the order of clinical exams was identical for all patients, starting with the MMP-9 immunoassay which was followed by Schirmer 1 test, biomicroscopic examination of the TBUT, corneal and conjunctival fluorescein staining, and lastly examination of the lid margins and meibomian glands [24]. After completing the series of physical exams, patients filled out the OSDI questionnaire and ocular irritation symptom score.

### Lid debris debridement combined with meibomian gland expression (MGX)

Patients underwent one session of BlephEx treatment with MGX performed by a single physician (HL). For any patients already in use of topical or systemic medication for dry eye disease, a minimum of 4 week washout period was required before enrolling the study. The BlephEx system was designed to exfoliate the eyelid margin with a rapidly spinning sponge-tipped microbrush and a foam cleanser that is swept back and forth along the lid margin for approximately 1–2 min to remove the bacterial biofilm, lid debris, and *Demodex folliculorum* mites [18, 27, 28]. The procedure is completed with 4 disposable brushes (1 for each eyelid margin) after removal of all visible lid scurf and cylindrical dandruff. Immediately after BlephEx treatment, meibomian gland of both upper and lower eyelids of each eye was expressed using a meibomian gland forcep after administering topical 0.4% oxybuprocaine hydrochloride as anesthetics. Daily lid hygiene regimen using an eyelid scrub and warm compression was recommended to all patients in addition to use of 0.18% sodium hyaluronate (Kynex2, Alcon Laboratory, Seoul, Korea) on demand during the follow-up period.

### Statistical analysis

Data were analyzed using linear mixed model, with AR (1) correlation structure. Generalized estimating equations model was used to compare non-continuous variables including ocular irritation symptom score, mucocutaneous junction replacement, meibum grade, and MGD stage. Linear mixed model and χ^2^ test were used to analyze MMP-9 immunoassay results. Statistical analysis was performed using SPSS software version 25.0 (IBM, Armonk, NY, USA). A *P* value of less than 0.05 was considered statistically significant.

## Results

The mean age of the patients was 63.1 ± 10.6 (range, 46–86) years. The majority of the subjects was women (71%). Table 1 shows the changes in the clinical findings and symptoms before and at 4 weeks after lid debris debridement and MGX. A significant increase in the TBUT (*P* = 0.002), along with improvement in SICCA ocular staining score (*P* < 0.001) and Oxford staining score (*P <* 0.001) were observed at 4 weeks after the treatment. However, there was no significant difference in the Schirmer test results (*P* = 0.703). Significant improvement in mean OSDI score (*P* = 0.002) and ocular irritation symptom score (*P* < 0.001) indicates that there is a notable alleviation of symptoms. Significant improvement in lid margin abnormality, including telangiectasia, plugging, and thickness was detected at 4 weeks after the treatment (Table 2). There was a significant decrease in the lid margin telangiectasia, plugging, and thickness scores in both the upper and lower eyelids (all *P* < 0.001). However, we did not observe a significant change in the lid margin irregularity score and mucocutaneous junction replacement.

**Table 1:** Clinical signs and subjective symptoms before and after eyelid debris debridement and meibomian gland expression in patients with moderate and severe meibomian gland dysfunction

Parameters	Before treatment	4 weeks after the treatment	*P* value
TBUT (sec)^a^	4.21 (0.30)	5.55 (0.21)	0.002
Fluorescein staining			
SICCA staining score^a^	4.72 (0.25)	3.17 (0.22)	< 0.001
Oxford staining score^a^	1.67 (0.10)	1.15 (0.09)	< 0.001
Schirmer test (mm)^a^	10.65 (1.32)	11.12 (1.49)	0.703
Subjective symptom			
OSDI score^a^	55.79 (3.18)	45.27 (2.98)	0.002
Ocular irritation symptom score^b^	33 (73.3%)	24 (53.3%)	< 0.001

*TBUT* tear film break-up time, *SICCA* Sjögren’s International Collaborative Clinical Alliance, *OSDI* ocular surface disease index

^a^Results are presented as mean (standard error) and *P* values are from linear mixed model

^b^Generalized estimating equations model for noncontinuous scale values: ocular irritation symptom score, n (%, proportion ≥ grade 2)

**Table 2:** Lid margin abnormality, meibomian gland function, and meibomian gland dysfunction stage before and after eyelid debris debridement and meibomian gland expression in patients with moderate and severe meibomian gland dysfunction

Parameters	Before treatment	4 weeks after the treatment	*P* value
Lid margin abnormality			
Lid margin irregularity^a^			
UL (0–2)	1.57 (0.07)	1.32 (0.08)	0.047
LL (0–2)	1.57 (0.07)	1.36 (0.09)	0.117
Telangiectasia^a^			
UL (0–3)	1.96 (0.07)	1.37 (0.10)	< 0.001
LL (0–3)	1.96 (0.07)	1.43 (0.10)	< 0.001
Meibomian gland orifice plugging^a^			
UL (0–2)	1.79 (0.07)	1.05 (0.09)	< 0.001
LL (0–2)	1.75 (0.09)	1.09 (0.09)	< 0.001
Thickness^a^			
UL (0–2)	1.60 (0.07)	1.02 (0.08)	< 0.001
LL (0–2)	1.54 (0.07)	1.07 (0.09)	< 0.001
Mucocutaneous junction replacement^b^	26 (56.5%)	18 (40.9%)	0.115
Meibum characteristics			
Meibum color^a^			
UL (1–3)	1.92 (0.07)	1.66 (0.09)	0.026
LL (1–3)	1.96 (0.09)	1.48 (0.08)	< 0.001
Meibum consistency^a^			
UL (1–3)	1.69 (0.09)	1.25 (0.07)	< 0.001
LL (1–3)	1.65 (0.07)	1.23 (0.06)	< 0.001
Meibum grade^b^			
UL (0–3)	38 (79.2%)	12 (25.0%)	< 0.001
MGD stage^b^	39 (84.8%)	10 (21.7%)	< 0.001

*MGD* meibomian gland dysfunction, *UL* upper lid, *LL* lower lid

^a^Results are presented as mean (standard error) and *P* values are from linear mixed model

^b^Generalized estimating equations model for noncontinuous scale values: mucocutaneous junction replacement, n (%, proportion for positive result), meibum grade, n (%, proportion ≥ grade 2), MGD stage, n (%, proportion ≥ stage 3)

**Fig. 1:**
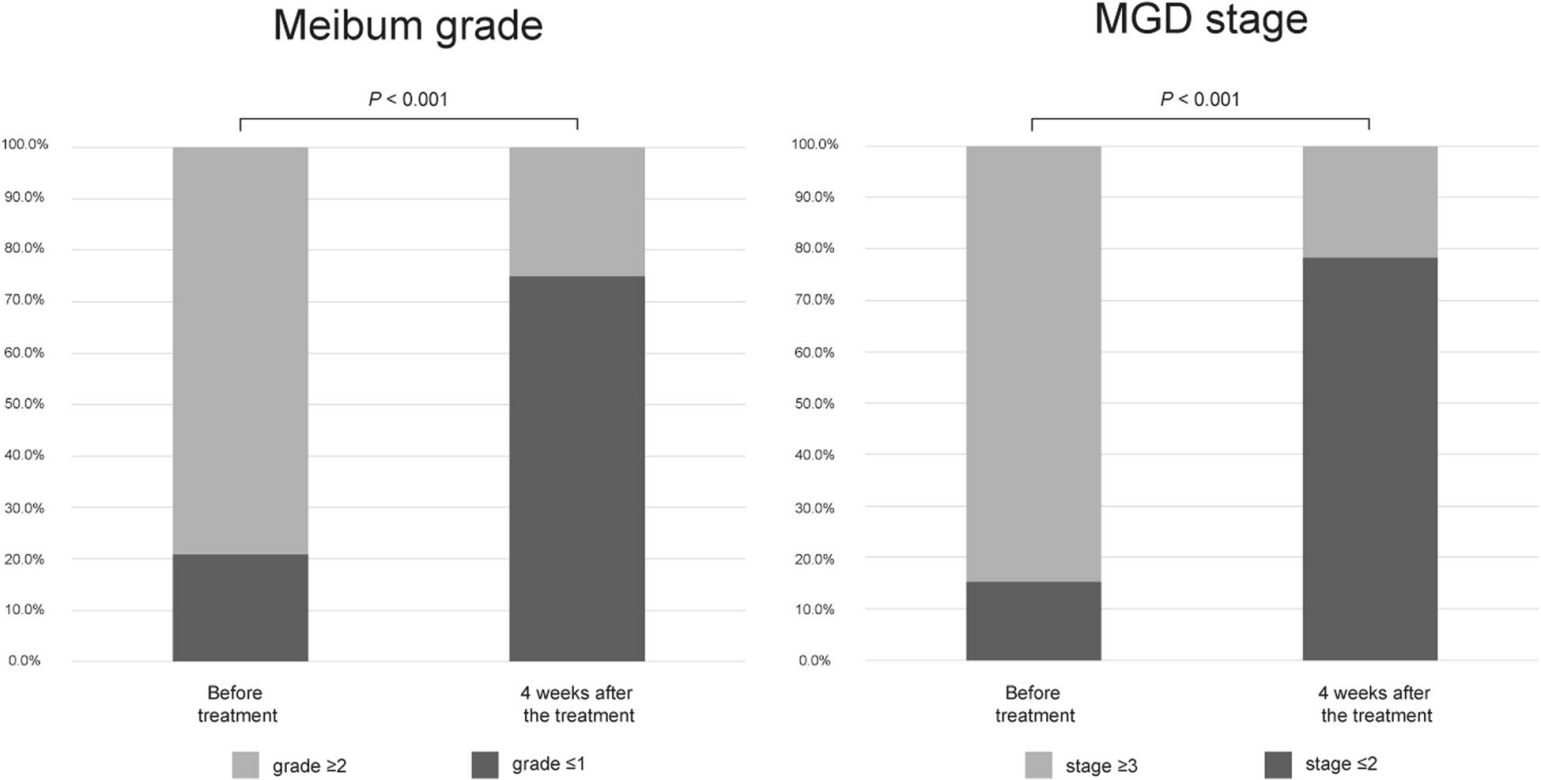
Change in the proportion of eyes of meibum grade 2 or 3 (left) and the proportion eyes of meibomian gland dysfunction stage 3 or 4 (right) before and after eyelid debris debridement and meibomian gland expression. MGD = meibomian gland dysfunction

Notable improvements in the meibomian gland function in both the upper and lower eyelids were observed in terms of meibum color (*P* = 0.026 for the upper eyelid and *P* < 0.001 for the lower eyelid) and meibum consistency (*P* < 0.001 for the upper and lower eyelids) (Table 2). Accordingly, the proportion of eyes of MGD grade 2 or 3 decreased significantly (*P* < 0.001). Finally, the proportion of eyes with MGD stage 3 or 4 showed a significant reduction (*P* < 0.001) (Fig. 1).

**Fig. 2:**
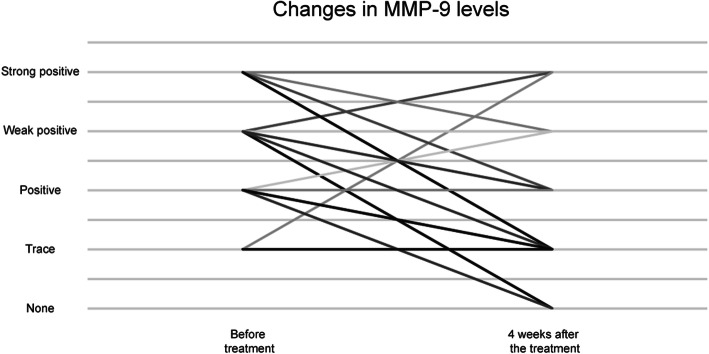
Changes in matrix metalloproteinase-9 levels before and after eyelid debris debridement and meibomian gland expression in each patient. MMP-9 = matrix metalloproteinase-9

Significant improvement in the mean ocular surface MMP-9 level (*P* = 0.029) was observed measured based on a scale of 0 to 4 for intensity of color (0, none; 1, trace; 2, weak; 3, positive; and 4, strong positive) (Table 3). Changes in MMP-9 level before and after eyelid debris debridement and MGX in each patient are shown in Fig. 2. In additional analysis, MMP-9 positivity rate significantly decreased (*P* = 0.014) at 4 weeks after the treatment (12 out of 24 cases; 50.0%) compared to pre-treatment (20 out of 24 cases; 83.3%). There was no allergic reaction and mechanical erosion observed post-treatment.

**Table 3:** Changes in matrix metalloproteinase-9 levels before and after eyelid debris debridement and meibomian gland expression in patients with moderate and severe meibomian gland dysfunction

Parameters	Before treatment	4 weeks after the treatment	*P* value
MMP-9 levels^a^	2.58 (0.20)	1.92 (0.26)	0.029
MMP-9 positivity^b^	20 (83.3%)	12 (50.0%)	0.014

*MMP-9* matrix metalloproteinase-9

MMP-9 levels were measured based upon the changes in the intensity of color on a scale of 0 to 4, as follows: 0, none; 1, trace; 2, weak positive; 3, positive; and 4, strong positive

^a^Results are presented as mean (standard error) and *P* values are from linear mixed model

^b^χ^2^ test: n (%, proportion for positive result: weak, positive, and strong positive)

## Discussion

In the present study, we demonstrated that combined treatment of lid debris debridement using the BlephEx and MGX significantly improved clinical findings, subjective symptoms, meibomian gland functions, and MMP-9 level in patients with moderate to severe MGD, advocating the use of this treatment combination as an effective strategy in treatment of moderate to severe MGD.

Eyelid debris debridement using the BlephEx was developed in the belief that the bacterial biofilm along the eyelid margin is responsible for meibomian gland inflammation [28]. Nattis et al. suggested that bacteria colonize the eyelid margin within a structure known as a biofilm, which causes eyelid inflammation, leading to MGD and aqueous insufficiency. Untreated MGD will inevitably result in eyelid destruction [29]. Normal bacterial floras, which are mainly *Staphylococcus epidermidis* and *Staphylococcus aureus*, living in the conjunctival sac and eyelid margin produce a biofilm that accumulates over many years [30]. When the population of *Staphylococcus* reaches a certain density, the quorum-sensing genes are activated by chemical signaling between organisms using homo-serine lactones and ion channels [31]. In turn, this gene activation results in the production of toxins and virulence factors that attack the host tissue to facilitate the expansion of the over-crowded biofilm [32, 33]. Based on those reports suggesting the role of biofilm as a virulence factor in the ocular surface inflammation, routine mechanical removal of the biofilm on the eyelid margin cannot be more emphasized in prevention of development and aggravation of ocular surface inflammation. Consequently, the use of BlephEx, a convenient tool for eyelid debris debridement, was introduced and has been applied in clinical settings as an effective way to treat eyelid and ocular surface inflammation [18].

In our study, eyelid debris debridement combined with MGX yielded a significant improvement in TBUT, SICCA ocular staining score, Oxford staining score, MGD stage, and OSDI questionnaire score at 4 weeks after treatment. In one recent study evaluating the effect of Cliradex terpinen-4-ol medicated lid scrubs and micro-blepharoexfoliation using the BlephEx for the treatment of *Demodex* blepharitis, this combination of treatment showed a statistically significant reduction in *D. folliculorum* infestation levels [18]. However, there was no significant improvement in OSDI score, tear osmolarity, extracellular MMP-9 levels, Schirmer 1 test, lid margin appearance, meibomian gland dropout, and meibomian gland secretions. Previous attempts to treat blepharitis associated with *D. folliculorum* include tea tree oil lid scrubs, oral and topical ivermectin, and oral metronidazole, but with varying reported success rates [34, 35]. Additionally, *demodex* blepharitis is usually accompanied by meibomian gland dropout and cicatricial eyelid remodeling, thus making treatment more difficult. In our study, based upon relatively poor response of micro-blepharoexfoliation using the BlephEx in the treatment of *Demodex* blepharitis, we aimed to evaluate the effects of eyelid debris debridement combined with MGX in the treatment of moderate and severe MGD.

We demonstrated a significant improvement in the mean value of MMP-9 levels (*P* = 0.029) and proportion of patients showing positive result for MMP-9 levels (*P* = 0.014) at 4 weeks after the treatment (50.0% after treatment versus 83.3% before treatment). This finding is consistent with previous study, which showed that an improvement in meibum expressibility positively correlated with the reduction of the tear inflammatory cytokines [36]. Ocular surface inflammation stimulates MMP-9 production by corneal epithelium and fibroblasts. Elevation of MMP-9 level on ocular surface in turn escalates production of other inflammatory cytokines and mediators exacerbating the chronic inflammatory cycle [37]. Elevation of MMP-9 level is associated with poor epithelial healing, which is also responsible for ocular surface inflammation and dry eye [25, 38]. As demonstrated previously, tear film MMP-9 level is a reliable quantitative index for ocular surface inflammation and tends to increase proportionally with the severity of dry eye. Its activity was significantly elevated in patients with MGD as well [39, 40]. MMP-9 immunoassay device is a rapid and precise tool that can measure MMP-9 in tear film with a sensitivity of 85%, a specificity of 94%, a negative predictive value of 73%, and a positive predictive value of 97% in diagnosing dry eye [25, 41]. We evaluated the correlation between MMP-9 level and clinical findings using Spearman rank-order correlation which showed a statistically significant association. Increase in MMP-9 level was associated with decrease in TBUT (*P* = 0.026, *R*^*2*^ = -0.338), increase in SICCA staining score (*P* = 0.001, *R*^*2*^ = 0.451), increase in Oxford staining score (*P* = 0.001, *R*^*2*^ = 0.497), and exacerbation of MGD grading (*P* = 0.009, *R*^*2*^ = 0.468) [42].

Meibum characteristic also showed statistically significant improvement after use of BlephEx followed by MGX. One possible explanation for this would be the removal of biofilm which subsequently reduces lipase-producing bacterial burden on the eyelid [43]. Bacterial lipase can affect the composition of meibum, which in turn can change the composition of the lipid layer. This disruption of homeostasis results in tear film instability and ocular surface inflammation [28, 30, 44, 45]. The blockage of meibomian gland ducts further induces bacterial proliferation, which facilitates the production of more lipase and finally induces a vicious cycle [46–48].

Lid margin abnormalities such as telangiectasia, meibomian gland plugging and thickness significantly improved at 4 weeks after the treatment. However, lid margin irregularity and mucocutaneous junction replacement showed no significant improvement. Lid margin telangiectasia, which is associated with vascular dilatation due to inflammatory mediators, and meibomian gland orifice plugging, which is related to altered meibum composition, are two features of dry dye disease that are relatively easier to reverse [46]. However, lid margin irregularity and mucocutaneous junction replacement, which develop over time due to chronic inflammation, are structural changes that take more time and are difficult to reverse into a normal structure [1, 19, 48]. Thus, it would only be fair to predict that more than one session of lid scrubbing with the BlephEx is required in order to detect any positive change in eyelid margin irregularity and mucocutaneous junction. As this study evaluated the effect of one session of lid debris debridement and MGX in treating moderate and severe MGD, a further study to assess the efficacy and safety of extended session of lid debris debridement and MGX seems necessary.

This study is limited by a small number of patients and its retrospective nature. Secondly, the duration of follow-up was limited to 4 weeks after treatment. Given that the follow-up period after one session of treatment was short, further investigation is needed to assess the long-term effectiveness and safety of multiple rounds of lid debridement. And prospective randomized controlled study with proper sample size is also necessary. It would also be helpful to evaluate the change of additional tear cytokines and composition of meibum profile after eyelid margin debridement in order to expand our understanding in the mechanism of dry eye disease.

## Conclusions

We have shown that one session of combination of eyelid debris debridement using the BlephEx and MGX is an effective treatment modality for patients with moderate and severe MGD in terms of improving patient symptoms and clinical signs, and reducing level of MMP-9 in treated patients. We believe that the improvement in signs and symptoms could be attributed to decreased MMP-9 level detected after treatment. Further study is mandatory to evaluate the efficacy and safety of extended session of lid debris debridement and MGX.

## Data Availability

Data are available upon request from the authors.
